# Compliance of private pharmacies in Uganda with controlled prescription drugs regulations: a mixed-methods study

**DOI:** 10.1186/s13011-020-00261-x

**Published:** 2020-02-18

**Authors:** Pakoyo Fadhiru Kamba, John Mulangwa, Bruhan Kaggwa, Freddy Eric Kitutu, Nelson Kaulukusi Sewankambo, Elly Tebasoboke Katabira, Pauline Byakika-Kibwika, Richard Odoi Adome, Robert Cyril Bollinger

**Affiliations:** 1 Department of Pharmacy, College of Heath Sciences, Makerere University, P. O Box, 7072 Kampala, Uganda; 2 Department of Internal Medicine, College of Heath Sciences, Makerere University, P. O Box, 7072 Kampala, Uganda; 3 School of Medicine, Johns Hopkins University, 600 N. Wolfe Street, Baltimore, MD 21287 USA

**Keywords:** Controlled prescription drugs, Compliance predictors, Dispensing practices, Stock control practices, Opioids, Controlled drug regulations

## Abstract

**Background:**

Controlled prescription drug use disorders are a growing global health challenge in Sub-Saharan Africa. Effective supply chain regulations on dispensing and stock control are important for controlling this epidemic. Since compliance with these regulations in resource-limited countries is poor, there is need to understand its predictors in order to reduce the risk of prescription drug use disorders.

**Methods:**

A mixed-methods study utilizing a structured questionnaire and a simulated client guide was undertaken in Kampala and Mbale towns in Uganda. The questionnaire recorded self-reported dispensing and verified stock control practices and their covariates from 101 private pharmacies. The guide recorded actual dispensing practices from 27 pharmacies. Snowball sampling was done to enrich the sample with pharmacies that stock opioids. The mean compliance with good dispensing and stock control practices was calculated. Multivariate logistic regression analyses were applied to identify predictors of compliance.

**Results:**

The mean compliance with dispensing and stock control requirements was 82.9% and 23%, respectively. Twenty percent and 40% of the pharmacies dispensed pethidine without a prescription and with invalid prescriptions, respectively. Having a pharmacist on duty (OR = 5.17; *p* = 0.02), prior in-service training on narcotics regulations (OR = 3.51; *p* = 0.04), and previous narcotics audits by the regulator (OR = 5.11; *p* = 0.01) were independent predictors of compliance with stock control requirements. Pharmacies with a previous history of poor compliance with dispensing requirements were less likely to demonstrate good compliance (OR = 0.21; *p* = 0.01).

**Conclusions:**

There is suboptimal compliance to controlled prescription drug regulations among Uganda’s pharmacies. A previous history of poor compliance to dispensing requirements predicted low compliance in subsequent assessments. Training and regulatory audits increased compliance in stock control but not dispensing. Expansion of training and audits to more pharmacies and/or incentives for compliance are necessary.

## Background

There is a growing global problem of controlled prescription drug (CPD) use disorders. The prescription opioid use disorder crises in the United States (U.S), Canada and Eastern Europe have drawn the most attention [1–6]. However, recent trends in Africa and Asia suggest CPD use disorders may be rapidly expanding. In Nigeria, a recent report indicates that 4.7% of its population (or 4.6 million people) indulged in non-medical use of prescription opioids in the past year, comprising 32% of all non-medical drug use in the country and lagging behind only cannabis use [7]. Furthermore, 2.4% of Nigeria’s population (or 2.4 million people) engaged in non-medical use of over-the-counter cough syrups containing opioids in the past year [7]. In addition, West and North Africa have witnessed an unprecedented rise in non-medical use and trafficking of tramadol, a prescription opioid not yet scheduled under international conventions [1, 8]. Similarly, a rise in methamphetamine use disorder has been reported in South Africa [9]. On the other hand, increased diversion and non-medical use of the lower potency prescription opioids buprenorphine, codeine, nalbuphine and dextropropoxyphene in Asia have been reported [10].

Controlled prescription drug use disorders are associated with devastating health effects, including fatal overdose, accidents, suicide, unwanted pregnancies, blood borne infections, cardiovascular complications and severe mental disorders [9, 11–13]. Hence, it is essential to prevent inappropriate use of CPDs. Accordingly, all nations are obliged to control availability of CPDs to the population in such a way that only individuals that need them for legitimate medical indications under a valid prescription access them [14–16]. In Uganda, national laws founded on international conventions restrict the power to prescribe CPDs to authorized medical, dental and veterinary practitioners, and the authority to dispense these drugs to licensed pharmacies under the direct supervision of a pharmacist [17, 18]. Uganda’s law also requires that CPDs are only dispensed with a valid prescription from an authorized prescriber [17]. In addition, pharmacies are required to keep dispensing and wholesale records for stock control and accountability, including drug returns to the Medicines Regulatory Agency (MRA). Without adherence to appropriate dispensing practices, identification and control of illicit purchases from pharmacies is difficult. Similarly, without appropriate stock control practices, accountability for CPDs is difficult.

Despite global solidarity on CPD control, leakages of CPDs from the legitimate supply chain remain of concern, even in countries with strong institutional systems such as the U.S. In the U. S, diversion of these drugs from doctor’s premises, pharmacies, hospitals, and legitimate patients has been implicated as the primary source for both drug dealers and unlawful consumers [19–22]. Research on U. S drug dealers has revealed that they obtain their CPD stocks primarily through pain clinic/doctor shopping either directly or through sponsored patients, purchases from legitimate patients or barter exchange with illicit drugs, undercounting of dispensed medications and falsification of inward inventories by pharmacy staff, and theft of prescription pads [22]. It has further been reported that unlawful CPD consumers also combine sourcing from illicit market suppliers with direct diversion from doctor clinics, pharmacies, hospitals, and lawful consumers among their family, friends and school peers [20, 21]. Though it occurs, cross-border smuggling of CPDs from the Caribbean and Latin America into the U. S lags behind inland diversion from the lawful supply chain [20].

International treaties on narcotics control allow compound analgesics and cough syrups containing reduced strengths of weak opiates such as codeine and dextromethorphan alongside non-opiates to be dispensed to customers without a prescription [14, 16, 23]. Consequently, compound opioid pharmaceutical preparations are available in pharmacies and other medicine outlets over-the-counter (OTC) in many countries [24]. Compound codeine products are permitted OTC in 13 out of the 28 European Union member states [25, 26] and in many other countries, including Australia [24], India [27], South Africa [28], and Uganda [17, 18] . The U. S permits sale of OTC dextromethorphan products [28]. Availability of compound opioids OTC is premised on the belief that reduced strengths of weak opioids are safe and carry negligible risk of triggering drug use disorders. However, non-medical use of OTC opioid products obtained from legitimate pharmaceutical retail outlets has been documented in many countries [28]. A recent review observed that countries in which these products have OTC status seemed most affected [28]. A study in Australia also found that OTC codeine dependent persons source their supplies from pharmacies, particularly those they considered less vigilant [24].

Thus, diversion of CPDs and their OTC forms from the legitimate supply chain is of significant importance in the dynamics of non-medical use, and interventions to minimize it are essential. For the CPDs, policies, practices and interventions to strengthen compliance of lawful suppliers with supply chain regulations is pertinent. In Uganda, the level of adherence to CPD supply regulations, such as those on appropriate dispensing and stock control is not known, although violations to pharmaceutical regulations have been reported [29]. Understanding the compliance to regulations on dispensing and stock control of CPDs in Uganda could inform strategies to optimize regulatory solutions, improve supply chain professional practices, and reduce the risk of drug use disorders in the country. Therefore, we determined the level and predictors of compliance to good dispensing and stock control requirements, among private pharmacies in Uganda.

## Methods

### Study design

A sequential mixed-methods research study was done to assess compliance with good dispensing and stock control practices of CPDs among private pharmacies in Uganda. Specifically, self-reported questionnaire survey data on compliance to prescription requirements during dispensing of CPDs was triangulated with simulated client data on actual dispensing practices from a subset of the survey sample during data interpretation and results discussion.

### Identification of eligible pharmacies

This study was conducted in Kampala Capital City and Mbale Municipality, the commercial centres for two of the four geographical regions of Uganda. In March 2018, Kampala and Mbale had 1029 private pharmacies with valid operating licenses from Uganda’s MRA, of which 622 were retail (licensed to dispense small quantities to patients only), 175 were wholesale (licensed to sell in bulk to retailers only) and 232 were dual (licensed for both wholesale and retail supply) [30]. There were two components in this study; 1) A questionnaire survey of compliance with dispensing and stock control requirements from dispenser self-responses and physical inspections, respectively; 2) A simulated client study to validate the self-reported dispensing practices for pethidine injection. For the questionnaire survey, snowball sampling in which the first pharmacies visited for data collection referred research assistants to other pharmacies likely having pethidine stocks was done. Purposive sampling is a method that prioritizes study units having the information of interest [31]. Therefore, for the simulated client sub-study, purposive sampling in which only pharmacies that acknowledged presence of pethidine in their premises in the self-report survey were selected was done.

For the questionnaire survey, a sample of 101 private pharmacies in Kampala in Central Uganda (*n* = 96) and Mbale in Eastern Uganda (*n* = 5) were selected. This sample size was adapted from the WHO guidelines on monitoring and evaluating country pharmaceutical situations which prescribes a minimum sample of 30 private outlets for a homogenous population [32]. Since this study involved three types of pharmacies (clusters), the sample was adjusted with a calculated design effect of 3.36 to account for inter-cluster variation in the population to yield an optimal sample of 101. For the simulated client sub-study of compliance to dispensing requirements for pethidine injection, the target sample was all the 28 pharmacies that acknowledged presence of pethidine stocks in their premises during the self-report sub-study. However, 27 of these pharmacies were actually studied as one was missed during assignment of simulated clients.

#### Recruitment for the questionnaire survey

Pharmacies were recruited for participation in the study with the aim of enriching the sample with those that stock pethidine injection. First, the MRA register of drugs as of March 2018 (Microsoft Excel format shared courtesy of NDA) was examined to identify authorized importers of pethidine injection. The importers of pethidine injection were then visited for data collection. Importers then linked the data collectors to other pharmacies that distribute and/or retail pethidine. From these leads, pharmacies in Kampala’s business suburbs proximal to large public and non-profit referral hospitals (Mulago, Nsambya, Mengo, Rubaga, Kawempe, Kiruddu, Butabika), those in suburbs with high night activity, those near large universities (Makerere University, Kyambogo University, Makerere University Business School, Ndejje University Kampala campus), and those on William street, an area of high pharmacy density in downtown Kampala were identified as suitable participants. Seven trained data collectors were then assigned to a different suburb/street from among these for data collection. For each data collector, the first accessed pharmacy willing to participate in the study was recruited, assessed and utilized for linkage to the next pharmacies in the locality with high likelihood of having pethidine stocks until a sample of 96 was obtained. For Mbale Municipality, pharmacies proximal to Mbale regional referral hospital and those on Republic Street, an area of high pharmacy density were identified as suitable participants. One willing pharmacy each on Republic street and proximal to the hospital was then assessed by two data collectors. Leads from these pharmacies were then used to recruit the remaining three pharmacies to make the stratum of five for this sub-study.

#### Recruitment for the simulated client sub-study

Pharmacies were recruited from among those that participated in the questionnaire survey and consented to participation in a subsequent simulated client study. All pharmacies that indicated that they do not stock pethidine injection during the questionnaire survey were excluded from the simulated client study.

### Data collection

#### Compliance to good dispensing and stock control practices

Information was obtained from personnel who dispense medicines in the pharmacies using a paper-based interviewer-administered structured questionnaire. In Uganda, these comprise pharmacists, pharmacy technicians, nurses and allied health professionals. Only dispensers who had worked at the pharmacy for at least 3 months were eligible for interview to ensure respondents had good understanding of the operations of the pharmacy. Prior to interviewing the dispenser, permission was obtained from the pharmacy manager using a written request. Then a trained data collector administered the consent for both this study and a future simulated client study to the dispenser.

The questionnaire was then administered to the consenting dispenser. The section on CPD dispensing collected only self-reported data; that is, dispenser responses were not verified for truths. Specifically, a dispenser was asked questions on whether the pharmacy stocks specific CPDs and whether they dispense certain controlled prescription drugs without a prescription. A tracer list of 7 representative drugs was used in this assessment. For CPD stock control, dispensers were asked about availability of specific records and written standard operating procedures (SOPs) for these drugs. Affirmative responses were verified through physical inspection by the interviewer. Twenty questions of which 10 applied to all CPDs and 10 were specific to particular drugs were used in the assessment.

#### Validation of self-reported compliance with prescription requirement in dispensing of CPDs

Pharmacies that acknowledged presence of pethidine in their premises were visited 6–8 weeks later by three forms of simulated client to validate compliance to the requirement of a prescription in dispensing of the drug. The three forms of simulated client were: client without a prescription, client with an invalid prescription, and client with an invalid bulk purchase order. To minimize the Hawthorne effect of consenting to the simulated client study on subsequent behavior of the dispensers, three strategies were used. Firstly, only a general statement “At a later time, other members of our research team will come to this pharmacy as customers so as to experience and document the actual practice” was used in the consent form. Secondly, simulated clients visited 4–8 weeks after the participant consent, which is sufficient for the Hawthorne effect to wane off. Thirdly, the simulated clients behaved as genuine customers; that is, they always had money to buy the pethidine and bought the drug where the dispenser availed it. Lastly, for each pharmacy, a different individual played each form of simulated client to avoid losing their anonymity, and each visited on a separate day. Each pharmacy was first visited by a simulated client without a prescription followed by a simulated client with an invalid prescription the next day. On the third day, wholesale pharmacies were visited by simulated clients with invalid bulk purchase orders. The simulated clients were the seven research assistants who conducted the preceding questionnaire survey. However, an individual was only assigned as a simulated client to pharmacies he/she had not visited in the questionnaire survey. A six-item, structured simulated client guide was used to record the actual dispensing practice by the data collector after exiting the pharmacy to a location out of its sight.

### Key study outcomes

This study had two study outcomes: 1) Compliance of pharmacies with the requirement of a prescription in dispensing of CPDs; 2) Compliance of pharmacies with stock control requirements for CPDs.

The compliance of pharmacies with the requirement of a prescription in dispensing of CPDs was assessed in two ways: 1) Self-reported compliance with the requirement of a prescription in dispensing of CPDs; 2) Validated compliance of pharmacies with the requirement of a prescription in dispensing of pethidine injection from simulated client data. Pethidine was a suitable CPD to validate the compliance for two reasons; a) it is a very strong opioid that quickly induces dependence and should be strictly kept away from non-medical use; b) it is widely used legitimately in the medical management of surgical pain, hence is more likely to be stocked by the legal private supply chain (pharmacies) than other strong opioids and stimulants. For the CPD stock control requirements, a validated compliance of pharmacies was obtained through physical verification of records and SOPs during the questionnaire assessment.

Meanwhile, the questionnaire survey also collected data on predictors of compliance; that is, factors likely to influence compliance with prescription and stock control requirements to enable examination of their association with compliance. These factors included pharmacy characteristics, dispenser characteristics, and regulatory supportive activities by the MRA. These variables were adapted from literature on non-compliance with pharmaceutical supply chain regulations for prescription only medicines and on diversion of opioids. Previous studies have found association between some dispenser characteristics and dispensing of prescription only medications without a prescription. Specifically, association of dispenser’s age, years of dispensing experience and professional qualifications with noncompliance with prescription requirements for antibiotics have been reported [33, 34]. A study on regulatory compliance of specialized drug shops in Kenya also found association between pharmacy qualifications and likelihood of regulatory compliance [35]. Meanwhile, findings from U. S studies indicate that opioids in the non-medical use market are primarily diverted from the legitimate supply chain as a result of regulatory and professional infringements [19–22].

Pharmacy characteristics examined were type of pharmacy, age of the business, and pharmacist presence. Dispenser characteristics examined were sex of dispenser, marital status of dispenser, dispenser’s profession, dispenser’s highest education level, and dispenser’s years of service at the pharmacy. Regulatory supportive activities examined were invitation to MRA workshops on narcotic drugs, MRA audits, MRA requests for narcotics returns, and previous MRA concerns on dispensing of narcotic drugs.

### Analyses

Data from the questionnaires and the simulated client guides was entered into EpiData 3.1 and cleaned. Transcription from EpiData into SPSS 13 was done for final cleaning, categorization of continuous data, computation of compliance and prevalence of compliant pharmacies, and bivariate analysis of predictors of compliance. Finally, data transcription from SPSS into STATA 12 was done for computation of adjusted odds ratios of the predictors of compliance by multivariate logistic regression.

#### Prevalence of pharmacies compliant with prescription and stock control requirements of CPDs

The self-reported prevalence of compliant pharmacies to the requirement of a prescription in dispensing of CPDs was calculated as the proportion of pharmacies that do not indulge in over-the-counter dispensing of these medications. Meanwhile, the prevalence of pharmacies compliant with the requirement of a valid prescription in dispensing of pethidine injection was calculated as the proportion of those that did not sell the drug to simulated clients who did not satisfy this requirement. Finally, the prevalence of pharmacies compliant with stock control requirements for CPDs was calculated as the proportion of those that had required stock records and SOPs.

#### Compliance of pharmacies with prescription and stock control requirements of CPDs

The composite self-reported compliance to the requirement of a prescription in dispensing of CPDs for each pharmacy (*Ci*) was measured as the percentage of compliant responses out of the seven survey questions assessed. From the composite compliance of each pharmacy, the mean compliance of all the pharmacies (*n* = 101) to the requirement of a prescription in dispensing of CPDs was then calculated using Eq (i).

i $$ Mean\ compliance\ of\ pharmacies\ to\ good\ practices=\frac{\sum_{i=1}^n Ci}{n} $$

The composite compliance to stock control requirements for each pharmacy was measured as the percentage of requirements that were satisfied out of the 20 survey questions assessed. From the composite compliance of each pharmacy, the mean compliance of all the pharmacies (*n* = 101) to stock control requirements of CPDs was also calculated using Eq (i).

#### Logistic regression of compliance with prescription and stock control requirements

Dispenser characteristics, pharmacy characteristics and regulatory factors were the explanatory variables for this analysis (details under Key Outcomes). A conceptual framework developed from literature guided the inclusion of these factors in logistic regression. Simple logistic regression was done to generate preliminary insights into the predictors of compliance to prescription and stock control requirements using chi square and unadjusted odds ratios. All these factors were then subjected to preliminary multivariate regression to determine the factors independently associated with compliance. Using backward elimination, those factors that had the weakest association were sequentially removed from the multivariate model, one at a time, until only those with *p*-values less than 0.5 were retained. Socio-demographic characteristics of the dispenser that showed strong statistical association with compliance were left in the multivariate regression models to control for confounding. For the prescription requirement, the minimum compliance score for pharmacies in the top quartile was used to delineate between low and high compliance categories for logistic regression. The cut-off for high compliance to prescription requirement was 100%. Similarly, the minimum compliance for pharmacies in the top quartile was used to delineate between low and high compliance to stock control requirements. The cut-off for high compliance to stock control requirements was 40%.

## Results

As shown in Table 1, the 101 pharmacies surveyed were predominantly retail in nature (59%). Medication dispensers at these pharmacies were primarily nursing professionals (66%) and had less than 1 year of experience in their current position (49%). Only 32% of the pharmacies had a certified pharmacist present on duty on the day of survey. Detailed raw data is found in Additional file 1.

**Table 1:** Characteristics of the pharmacies and dispensers who participated in the questionnaire survey

Characteristic	Category	Frequency n (%)
Type of pharmacy	Retail	60 (59.4)
	Wholesale (distributor)	22 (21.8)
	Dual (wholesale/retail)	19 (18.8)
Number of years pharmacy has been in operation	≤ 1 year	7 (7.0)
	2–3 years	24 (24.0)
	4–5 years	14 (14.0)
	> 5 years	55 (55.0)
Sex of dispenser	Male	45 (44.6)
	Female	56 (55.4)
Religious background of dispenser	Christian	82 (81.2)
	Muslim	18 (17.8)
	Hindu	1 (1.0)
Marital status of dispenser	Single	64 (63.4)
	Married	37 (36.6)
Dispenser’s qualifications	Pharmacist or intern pharmacist	8 (7.9)
	Pharmacy technician	17 (16.8)
	Nurse or midwife	67 (66.3)
	Other^1^	9 (8.9)
Dispenser’s highest education level	Secondary school	2 (2.0)
	Post-secondary school certificate	44 (44.9)
	Diploma (associate degree)	40 (40.8)
	Degree (Bachelors, Masters or doctorate)	12 (12.2)
Dispenser’s working experience at current work station	≤ 1 year	48.5
	2–3 years	27.7
	4–5 years	13.8
	> 5 years	10
Dispenser’s working experience since first qualification	≤ 1 year	16 (16.7)
	2–3 years	33 (34.4)
	4–5 years	18 (18.8)
	> 5 years	29 (30.2)
Pharmacist is present in the pharmacy premises on the day of data collection	Yes	32 (32.0)
	No	69 (68.0)

^1^Others include one Bachelor of Commerce graduate, one nursing assistant and 7 clinical officers (physician assistants)

For the simulated client analyses in 27 pharmacies, 18 were retail and 9 were wholesale. The simulated client analyses for the bulk sale of pethidine with an invalid purchase order that does not meet MRA requirement involved only the 9 wholesale pharmacies.

### Compliance with prescription requirements

As shown in Table 2, the proportion of pharmacies compliant with the requirement of a prescription in dispensing of different CPDs was less than 90% for all the drugs and was below 60% for diazepam and codeine tablets. Even the strong opioid pethidine was not spared in terms of OTC dispensing. The mean compliance of the 101 pharmacies to the requirement of a prescription in dispensing of CPDs, as derived from the composite compliance of each pharmacy was 82.9% (SD: 24.0%). Only 39.6% of the pharmacies scored a composite compliance of 100%.

**Table 2:** Prevalence of pharmacies that is compliant with the prescription requirement in dispensing of seven commonly used CPDs. The complement of each proportion gives the prevalence of pharmacies that are non-compliant

Dispensing practice	Sample size, N^1^	Frequency n (%)
Pharmacy only sells pethidine injection on a prescription	28	22 (78.6)
Pharmacy only sells phenobarbital tablets on a prescription	73	50 (68.5)
Pharmacy only sells diazepam tablets on a prescription	83	49 (59.0)
Pharmacy only sells ketamine injection on a prescription	33	29 (87.9)
Pharmacy only sells propofol injection on a prescription	11	9 (81.8)
Pharmacy only sells codeine tablets on a prescription	54	32 (59.3)
Pharmacy only sells tramadol injection on a prescription	92	59 (64.1)

^1^Only pharmacies which had a particular drug were analyzed for compliance

To gain further insights into OTC dispensing of CPDs, self-reported compliance to the prescription requirement in dispensing of pethidine injection was validated through a simulated client investigation. As shown in Table 3, this data confirmed that many pharmacies are not compliant with the requirement of a valid prescription in dispensing of these drugs.

**Table 3:** Prevalence of pharmacies that is compliant with the prescription requirement in dispensing of pethidine from self-reports versus simulated clients. The complement of each proportion gives the prevalence of pharmacies that are non-compliant

Source of data on pethidine dispensing practice	Sample size, N^1^	Frequency n (%)
Self-reported dispensing with no prescription	28	22 (78.6)
Dispensing to simulated client with no prescription	27	22 (81.5)
Dispensing to simulated client with ineligible prescription	27	17 (63.0)
Wholesale supply to simulated client with ineligible bulk purchase order	9	5 (55.6)

^1^Only the pharmacies that self-reported possession of pethidine stocks in the questionnaire survey were subjected to simulated client investigation

Ineligible prescriptions lacked complete prescriber’s full names, full address, telephone number and dose, as well as had inappropriate durations of treatment with pethidine injection. Ineligible purchase orders had concocted names of pharmacies and clinics, as well as lacked a purchaser’s stamp and authorization by the medicine regulatory agency.

### Compliance with stock control requirements

The mean compliance to stock control requirements for CPDs was 23.0% (SD: 21.5%). As shown in Table 4, the prevalence of pharmacies compliant with stock control requirements for CPDs was low. Even with a low threshold of 40%, only17.8% of the pharmacies scored in the high compliance category on stock controls. The prevalence of compliance to opioid stock control requirements was only 30%.

**Table 4:** Prevalence of pharmacies that is compliant with CPD stock control requirements. The complement of each proportion gives the prevalence of pharmacies that are non-compliant

Stock control requirement	Sample size, N	Frequency n (%)
Pharmacy has controlled prescription drugs book to record sales^1^	100	49 (49)
Controlled prescription drugs book documents batch number of dispended drug^2^	99	38 (38.4)
Controlled prescription drugs book documents telephone contact of customer^1^	100	21 (21.0)
Pharmacy has dedicated file for archiving copies of opioid prescriptions^1^	100	30 (30.0)
Pharmacy has dedicated file for archiving copies of psychotropic drug prescriptions	101	15 (14.9)
Pharmacy has dedicated file for archiving copies of authorized purchase orders of opioids^1^	100	40 (40.0)
Pharmacy has dedicated file for archiving copies of authorized purchase orders of psychotropic drugs^1^	100	20 (20.0)
Pharmacy has standard operating procedure for secure storage of prescription drugs^2^	99	40 (40.4)
Pharmacy has standard operating procedure for dispensing of prescription drugs^1^	100	39 (39.0)
Standard operating procedure for dispensing of prescription drugs is adhered to^3^	39	32 (82.1)
Pharmacy has stock card for pethidine injection^3^	28	13 (46.4)
Number of ampoules of pethidine injection in the stock card is equal to the physical count^3^	13	13 (100)
Pharmacy has stock card for methamphetamine tablets^3^	3	2 (66.7)
Number of methamphetamine tablets in the stock card is equal to the physical count^3^	2	2 (100)
Pharmacy has stock card for tramadol injection^3^	92	36 (39.1)
Stock of tramadol injection in the stock card is equal to the physical count^3^	36	34 (94.4)
Pharmacy has stock card for codeine tablets^3^	57	17 (29.8)
Stock of codeine tablets (unit packs) in the stock card is equal to the physical count^3^	17	16 (94.1)
Pharmacy has stock card for Fentanyl tablets^3^	4	4 (100)
Stock of Fentanyl tablets (unit packs) in the stock card is equal to the physical count^3^	4	3 (75.0)

^1^One questionnaire had missing data on this question. ^2^Two questionnaires had missing data on this question. ^3^These questions only applied to pharmacies that stocked a particular drug as established by this survey. Details are in Additional file 2

### Predictors of compliance with prescription and stock control requirements

The factors associated with compliance to prescription and stock control requirements were separately examined in order to inform strategies for optimizing CPD drug regulation in Uganda and other similar settings. After controlling for dispenser’s profession and years of service at the pharmacy, the only factor independently associated with compliance to the prescription requirement was history of suboptimal compliance during dispensing of narcotics in previous MRA inspections (Table 5). However, an inverse association was found, with pharmacies having a previous history of poor compliance less likely to demonstrate compliance in subsequent assessments.

**Table 5:** Predictors of compliance with the prescription requirement in dispensing of CPDs. Only regulatory factors remained in the model after controlling for confounding

Factor	Category	Frequency (n)		χ^2^	Crude OR (95% CI)	p-value	Adjusted OR (95% CI)	p-value
		High compliance	Low compliance					
MRA asked for returns of narcotic drug transactions^2^	Yes	24	14	0.19	1.21 (0.53–2.76)	0.659	2.45 (0.85–7.13)	0.095
	No	37	26					
MRA has ever audited narcotic drugs in pharmacy^2^	Yes	19	20	3.62	0.45 (0.20–1.03)	0.057	0.44 (0.16–1.23)	0.118
	No	42	20					
History of suboptimal compliance in storage of narcotics in previous MRA inspections^2^	Yes	24	20	1.12	0.65 (0.29–1.45)	0.291	2.24 (0.68–7.40)	0.185
	No	37	20					
History of suboptimal compliance in dispensing of narcotics in previous MRA inspections^2^	Yes	15	20	6.89	0.33 (0.14–0.76)	0.009	0.21 (0.06–0.73)	0.014
	No	46	20					

^1^Pharmacy professional comprises pharmacists and pharmacy technicians; non-pharmacy professional comprises nurses, nursing assistants, assorted health professionals such as clinical officers (physician assistants) and orthopaedic officers, and one accounting/finance professional. ^2^MRA, Medicines Regulatory Agency

After controlling for dispenser’s profession, years of service at the pharmacy and dispenser’s highest education level, three factors were independently associated with compliance to stock control requirements for CPDs. These were, pharmacist presence in the pharmacy, having ever been invited for a workshop on handling narcotics by the MRA, and having ever been audited for narcotics by the MRA (Table 6).

**Table 6:** Predictors of compliance with CPD stock control requirements. Only professional and regulatory factors remained in the model after controlling for confounding

Factor	Category	Frequency (n)		χ^2^	Crude OR (95% CI)	p-value	Adjusted OR (95% CI)	p-value
		High compliance	Low compliance					
Pharmacist is present in pharmacy premises	Yes	8	24	2.13	2.19 (0.75–6.33)	0.144	5.17 (1.30–20.57)	0.020
	No	9	59					
MRA has ever invited us for a workshop on handling of narcotic drugs^2^	Yes	12	23	9.91	5.22 (1.75–15.54)	0.002	3.51 (1.03–11.92)	0.044
	No	6	60					
MRA asked for returns of narcotic drug transactions^2^	Yes	13	25	11.17	6.03 (1.94–18.73)	0.001	1.93 (0.59–6.333	0.278
	No	5	58					
MRA has ever audited narcotic drugs in pharmacy^2^	Yes	12	27	7.27	4.15 (1.41–12.24)	0.007	5.11 (1.43–18.30)	0.012
	No	6	56					

^1^Pharmacy professional comprises pharmacists and pharmacy technicians; non-pharmacy professional comprises nurses, nursing assistants, assorted health professionals such as clinical officers (physician assistants) and orthopaedic officers, and one accounting/finance professional.^2^MRA, Medicines Regulatory Agency

## Discussion

There is a growing global problem of CPD use disorders. Compliance with supply chain regulations, especially pertaining to prescription and stock control requirements [14, 15, 36–38] is an important component of addressing the problem.

Contrasting values of compliance of Uganda’s private pharmacies with regulatory requirements for prescriptions and stock control were found. Whereas the mean compliance of the pharmacies with prescription requirements was found to be high (83%), that to stock control requirements was poor (23%). While this dissonance could be real, it may also be due to the contrasting approaches employed in collecting the compliance data. Compliance with prescription requirements was measured by dispenser self-reports whereas that with stock control requirements was verified through inspection of records and written SOPs. Self-reports are susceptible to social desirability bias that may lead to underreporting of sensitive events and over-estimation of favourable ones [39, 40]. Indeed, some studies of dispensing practices in pharmacies have reported discordance between self-reports and actual practices. In one such case in Hanoi (Vietnam), while a questionnaire self-report showed that 20% of pharmacies dispensed antibiotics for acute respiratory infections in children, 83% of these pharmacies were found to do so in a simulated client study [41]. However, our findings from a case study of compliance with prescription requirements for pethidine injection suggest that underreporting was minimal in this work. The prevalence of pharmacies compliant with prescription requirements in dispensing of pethidine injection was similar under self-report (79%) and simulated client (82%); that is, about 20% of the pharmacies are non-compliant. Overall, the proportion of non-compliant pharmacies with prescription requirements for CPDs varied from the 20% with pethidine to 41% with diazepam tablets. Studies of dispensing practices for other prescription only medicines (POMs) in sub-Saharan Africa and Asia, particularly antibiotics, have reported much higher proportions of non-compliant pharmacies than what we have found with CPDs. A study of 73 retail pharmacies in Zambia found that 100% dispensed antibiotics without a prescription [42]. In Uganda, a study of 170 registered drug shops found that 93.5% prescribed antibiotics over-the-counter [34]. In Tanzania a simulated client study of 85 accredited drug dispensing outlets (ADDOs) reported that 79% dispensed antibiotics without prescriptions [43] while in Hanoi, Vietnam, 83% of the pharmacies dispensed antibiotics without a prescription [41].

Although compliance of Uganda’s pharmacies with prescription requirements for CPDs is higher than that reported for other POMs it is inadequate as compliance by all pharmacies is necessary if diversion for non-medical use is to be avoided. Important lessons can be drawn from the easy availability of OTC compound formulations containing reduced strengths of weak opioids that has led to significant non-medical use of these medications in many countries [28], including those in Africa. In Nigeria, 2.4% of the population are engaged in non-medical consumption of opioid containing cough syrups [7]. Though illegal street vendors dominate the non-medical use tramadol market OTC sale of tramadol in pharmacies has also been mentioned among the drivers of index non-medical exposure to the drug in West Africa [8], which is a key milestone in the pathway to problematic drug use. Meanwhile, drug diversion is also a fairly lucrative and complex industry in which even a single opportunity for diversion can be maximized. Despite the stringent regulatory environment in the U.S, its CPD diversion industry is large (at least USD 25 billion annually), and key players (suppliers, brokers, consumers) exploit any little opportunity to siphon CPDs from the lawful supply chain [19, 20]. Insights from the U.S show that a major strategy used to divert large CPD volumes by illicit suppliers is targeted clearance of drug stocks from the few vulnerable pharmacies using contingents of sponsored patients [22]. Even a single opportunity for a vulnerable pharmacy can be deeply exploited for CPD diversion by the unlawful market. Therefore, a situation in which up to 40% of pharmacies in Uganda can supply CPDs without prescription presents a fertile ground for diversion. Whether the sizable opportunity of accessing of CPDs without a prescription translates into rampant CPD diversion in Uganda is unknown because data on actual diversion of CPDs in the country is lacking. Thus, further research is needed to elucidate the volume and monetary value of Uganda’s CPD diversion industry, as well as the mix of players involved.

Poor adherence to stock control requirements for CPDs, as found in this study, undermines accountability and creates opportunity for diversion of these drugs from licensed pharmacies to the illicit market. In India, diversion of prescription opioids from the legitimate supply chain to the illicit market has been reported to be the dominant source of these drugs for non-medical use [10]. Besides, lack of accountability and inadequate control of these drugs also encourages inappropriate dispensing practices, which further exacerbates the risk of exposure of the population to these drugs. Poor CPD dispensing and stock control practices by private pharmacies in Uganda leaves a window of ready access for individuals who need them for non-medical use. The impact of poor CPD pharmacy practices on Uganda’s society is not yet known, as there is no country data on the level of CPD use disorders, its socio-demographic segmentation and its impacts on public health, criminal justice and socio-economic wellbeing.

Assessing the predictors of compliance to proper dispensing and stock control regulations could inform strategies to optimize compliance. In this study, pharmacies with a previous history of poor dispensing practices for narcotics hardly reformed. Weak pharmaceutical regulation has been implicated in exacerbating illicit practices in licensed pharmacies in many low and middle income countries, including India [10] and East Africa [29, 44, 45]. Thus, there is need for strengthened regulatory support and/or incentives to encourage compliance. Furthermore, understanding the factors underlying persistent non-compliance among pharmacies that are repeatedly non-compliant with regulations is necessary if sustainable gains in CPD regulation are to be realized.

We found key professional and regulatory factors to be strong predictors of compliance to good stock control practices. Professionally, active pharmacist involvement in the operations of the pharmacy, here measured by pharmacist presence on duty in the pharmacy premises on the day of assessment, was essential to compliance. As the top professionals in pharmacies, pharmacists oversee all dispensing services and are accountable for regulatory and professional compliance.

As part of their work, pharmacists are responsible for validating CPD prescriptions with prescribing doctors and screening CPD clients to assess potential for non-medical use before dispensing. In a study of OTC codeine dependent individuals in Australia, participants averred that interactions with pharmacists often resulted in being denied the medication and that they preferred easy pharmacies where they face less scrutiny on drug use [24]. A study of how illicit drug suppliers in the U.S obtain their CPD inventories found that pharmacy fraud by pharmacy technicians in which they undercount dispensed medications and/or inward inventories is an important channel for diversion [22]. These reports, together with our findings suggest a need for vigilant pharmacist oversight over pharmacy assistants in dispensing of CPDs.

Whereas Uganda’s law states that both retail and wholesale pharmacy business shall be carried out under the immediate supervision of a pharmacist [17], the pharmacist in-charge was absent in most pharmacies on the day of assessment. A phenomenon in which most pharmacists only provide their practicing certificates to pharmacy owners for licensing and stay away from the pharmacy premises thereafter has been reported to be rampant in developing countries [44]. Given the inadequate pharmacist numbers in low income countries [46], MRAs and pharmacist professional regulatory agencies need innovative strategies to mitigate pharmacist absenteeism in pharmacies and its impact on compliance with medicines regulations. These strategies could entail strengthened MRA support supervision of pharmacies, continuing professional development (CPD) of dispensing staff, understanding disincentives to pharmacist availability in pharmacies, and recognition of the most compliant pharmacies, among others. There may also be need for strengthening of pharmacist professional regulation in Uganda to boost accountability and minimize professional and regulatory infringements in medication dispensing. Currently, oversight over pharmacists in Uganda is a convoluted process with multiple players. The pharmacy board that registers and disciplines pharmacists has no authority over pharmacy licensing, the MRA that licenses pharmacies has no authority over pharmacist discipline, and the professional association whose membership is a prerequisite to practicing pharmacy in Uganda has neither pharmacy licensing nor pharmacist disciplinary mandates [17, 47]. Without effective communication between these multiple players, weak pharmacist professional regulation ensues. Critically, there is need for legislative amendments to harmonize pharmacist professional regulation and pharmacies’ licensing by putting them under one roof, either under the body responsible for pharmacist membership (Pharmaceutical Society of Uganda) or registration (Pharmacy Board). This is the situation in some countries, including many of low income status [48].

Meanwhile, MRAs ought to scale up the various regulatory activities that were found independently associated with compliance to good stock control practices. Specifically, MRAs need to conduct regular training of pharmacy managers, owners and professional staff on laws, regulations and good practices in handling of narcotics and CPDs, as well as increase audits of pharmacies for these drugs. Regulatory agencies could also enforce regular inventory, prescription and drug dispensing reports by pharmacies to promote CPD accountability. Low income countries could also borrow a leaf from the U.S prescription drug monitoring programs (PDMPs) that have been employed in collecting, analyzing, disseminating and retrieval of CPD prescribing, dispensing, use and patient data to stakeholders and flagging suspect practices and repetitive customers [22, 49]. In this work, ineligible prescriptions increased the proportion of pharmacies that dispensed pethidine to the simulated clients. Thus, systems for pharmacies to validate doctor prescriptions for CPDs before dispensing to deter use of forged prescriptions are also necessary.

### Study limitations

The study utilized snowball sampling which is prone to various selection biases. Firstly, as a non-probability sampling method that relies on the subjective knowledge of the first study participants, snowball samples may not be representative of the whole population which limits the generalization of findings. Secondly, as a chain referral technique, snowball sampling tends to favour sampling units within the social networks of index participants resulting into cohesive samples enriched in certain characteristics not representative of the population [50]. Furthermore, chain referral can escalate response bias if information about the study leaks to downstream study participants within social networks [51]. However, snowball approach was appropriate for our study because stocking strong opioids was critical to the study outcomes yet a heterogeneous sampling frame comprising both pharmacies with and without these drugs was used. This study also utilized self-reports for the determination of predictors of compliance to good dispensing practices. Self-reports can underreport sensitive events and exaggerate favourable ones [39, 40]. Our validation of compliance to good dispensing practices through the simulated client investigation of actual dispensing practices, and our verification of stock control systems through physical inspection minimized this bias though. Indeed, the simulated client data of pethidine dispensing suggested that underreporting in the self-reports was low. Nevertheless, a holistic picture on barriers to compliance with good pharmacy practices for CPDs requires collection and synthesis of dispensers’ qualitative experiences with dispensing and stock control of these drugs.

## Conclusions

There is low compliance to regulations on CPDs among private pharmacies in Uganda. A history of low compliance to good dispensing practices of CPDs predicts subsequent non-compliance. Furthermore, pharmacist presence and regulatory supportive activities by the medicines regulator such as audits of CPD transactions and workshops on handling of CPDs predict compliance to good stock control but not dispensing practices among Uganda’s private pharmacies. This was the first scientific study to report on compliance to CPD regulations in Uganda. Not only does it provide a platform for further scientific exploration, it also provides useful evidence to inform policy and practice in the regulation of these drugs.

## Supplementary information

**Additional file 1.** Compliance survey dataset Microsoft Excel data set of the questionnaire survey redacted for potential participant identifiers to maximize confidentiality. The following data was deleted completely: type of pharmacy; nationality of dispenser; home region of dispenser; religious background; marital status.

**Additional file 2.** Prevalence of stocks of different controlled prescription drugs in Uganda’s private pharmacies Stocking practices of different CPDs among the pharmacies that participated in the questionnaire survey. Tramadol injection, tramadol capsules, diazepam, phenobarbitone, alprazolam, codeine, ketamine, and pethidine injection were the dominant CPDs stocked in Uganda’s pharmacies in decreasing order of prevalence.

## Data Availability

The datasets used and/or analyzed during the current study are available from the corresponding author on reasonable request.

## Abbreviations

CPDs: Controlled prescription drugs
GPPs: Good pharmacy practices
MRA: Medicines regulatory agency
OR: Odds ratio
OTC: Over-the-counter
SD: Standard deviation
SPSS: Statistical package for social sciences
