# Supplementary material for: Compliance of private pharmacies in Uganda with controlled prescription drugs regulations: a mixed-methods study
Source: Subst Abuse Treat Prev Policy. 2020 Feb 18;15:16. doi: 10.1186/s13011-020-00261-x (PMC7027211; doi:10.1186/s13011-020-00261-x)
Supplement: Supplementary file 2 — Additional file 2. Prevalence of stocks of different controlled prescription drugs in Uganda’s private pharmacies Stocking practices of different CPDs among the pharmacies that participated in the questionnaire survey. Tramadol injection, tramadol capsules, diazepam, phenobarbitone, alprazolam, codeine, ketamine, and pethidine injection were the dominant CPDs stocked in Uganda’s pharmacies in decreasing order of prevalence. [file 13011_2020_261_MOESM2_ESM.docx]

**Supplementary Table 1**. Prevalence of stocks of different controlled prescription drugs in Uganda’s private pharmacies

| Controlled prescription drug | Sample size, N | Frequency of pharmacies that stock the drug  n (%) |
| --- | --- | --- |
| Morphine injection | 101 | 15 (14.9) |
| Oral morphine | 101 | 1 (1.0) |
| Codeine tablets | 101 | 54 (53.5) |
| Oxycodone | 101 | 3 (3.0) |
| Hydrocodone | 101 | 4 (4.0) |
| Pethidine injection | 101 | 28 (27.7) |
| Tramadol injection | 101 | 92 (91.1) |
| Tramadol capsules or tablets | 101 | 92 (91.1) |
| Amphetamine | 101 | 7 (6.9) |
| Methamphetamine | 101 | 1 (1.0) |
| Ketamine | 101 | 33 (32.7) |
| Propofol | 101 | 11 (10.9) |
| Fentanil | 101 | 5 (5.0) |
| Alfentanil | 101 | 1 (1.0) |
| Sufentanil | 101 | 1 (1.0) |
| Oxycontin | 101 | 10 (9.9) |
| Phenobarbitone | 101 | 73 (72.3) |
| Amobarbital | 101 | 2 (2.0) |
| Secobarbital | 101 | 1 (1.0) |
| Diazepam | 101 | 83 (82.2) |
| Alprazolam | 101 | 57 (56.4) |
| Bromazepam | 101 | 17 (16.8) |
